# Transcriptome analysis uncovers the key pathways and candidate genes related to the treatment of *Echinococcus granulosus* protoscoleces with the repurposed drug pyronaridine

**DOI:** 10.1186/s12864-021-07875-w

**Published:** 2021-07-13

**Authors:** Yingfang Yu, Jun Li, Weisi Wang, Tian Wang, Wenjing Qi, Xueting Zheng, Lei Duan, Jiaxu Chen, Shizhu Li, Xiumin Han, Wenbao Zhang, Liping Duan

**Affiliations:** 1 NHC Key Laboratory of Parasite and Vector Biology, WHO Collaborating Centre for Tropical Diseases, National Institute of Parasitic Diseases, Chinese Center for Disease Control and Prevention, 200025 Shanghai, China; 2 State Key Laboratory of Pathogenesis, Prevention and Treatment of High Incidence Diseases in Central Asia, Clinical Medical Research Institute, the First Affiliated Hospital of Xinjiang Medical University, 830054 Urumqi, China; 3 Qinghai Provincial People’s Hospital, 810007 Xining, China

**Keywords:** *Echinococcus granulosus*, pyronaridine, RNA sequencing, Protoscoleces, MAPK, Heat shock protein, ABC transporter

## Abstract

**Background:**

Cystic echinococcosis (CE) is a life-threatening zoonosis caused by the larval form of *Echinococcus granulosus* tapeworm. Our previous study showed that an approved drug pyronaridine (PND) is highly effective against CE, both *in vitro* and in an animal model. To identify possible target genes, transcriptome analysis was performed with *E. granulosus sensu stricto* protoscoleces treated with PND.

**Results:**

A total of 1,321 genes were differentially expressed in protoscoleces treated with PND, including 541 upregulated and 780 downregulated genes. Gene ontology and KEGG analyses revealed that the spliceosome, mitogen-activated protein kinase (MAPK) pathway and ATP-binding cassette (ABC) transporters were the top three enriched pathways. Western blot analysis showed that PND treatment resulted in a dose-dependent increase in protein expression levels of *Eg*MKK1 (MKK3/6-like) and *Eg*MKK2 (MEK1/2-like), two members of MAPK cascades. Interestingly, several heat shock protein (HSP) genes were greatly downregulated including stress-inducible HSPs and their constitutive cognates, and some of them belong to *Echinococcus*-specific expansion of HSP70.

**Conclusions:**

PND has a great impact on the spliceosome, MAPK pathway and ABC transporters, which may underline the mechanisms by which PND kills *E. granulosus* protoscoleces. In addition, PND downregulates HSPs expression, suggesting a close relationship between the drug and HSPs.

**Supplementary Information:**

The online version contains supplementary material available at 10.1186/s12864-021-07875-w.

## Background

Cystic echinococcosis (CE) is a parasitic zoonosis caused by the larval stage of the dog tapeworm *Echinococcus granulosus.* Chronically infected humans or domestic animals remain asymptomatic for a long time. Infection with *E. granulosus* leads to the development of one or multiple cysts located mostly in the liver and lungs, which triggers clinical signs in the late stage, including abdominal and chest pain, chronic cough, vomiting, even death [1, 2]. CE is a globally distributed disease highly endemic in South America, Northern Africa, and Central Asia (especially Western China) [3]. The global health burden of CE is estimated at over 1 million disability-adjusted life-years (DALYs) each year [4]. The disease also affects the local livestock industry’s economic benefits, with an estimated yearly loss of at least US$3 billion [5].

Among current CE treatment options, anti-parasitic drug therapy is widely used for most clinical cases [6, 7]. Recently, we repurposed an approved anti-malarial drug pyronaridine (PND) as a promising candidate for CE treatment [8]. Oral administration of PND showed high concentrations of the drug in the liver and lungs, which are the most affected organs in CE. Oral administration, intraperitoneal injection or microinjection procedure (which mimics the clinical percutaneous techniques) significantly reduced the parasite burden in mice. However, the anti-CE mechanism of action of PND is not clear. Previous studies showed that the primary anti-malarial mode of action of PND is inhibition of β-hematin formation, enhancement of hematin-induced red blood cells lysis, and inducing the formation of abnormal vesicles in the food vacuole of plasmodium [9, 10]. A transcriptome profiling of *Plasmodium falciparum* in response to PND reveals a striking abundance of genes encoding host-exported proteins [11]. In addition, PND has been characterized as a potential anticancer agent, which reverses the multi-drug‐resistance (MDR) phenotype in MDR cancer cell lines by inhibiting the function of the efflux P‐glycoprotein (Pgp) [12, 13]. In this study, to obtain a comprehensive understanding of the anti-CE mechanism of PND, the global gene expression in *E. granulosus* protoscoleces (PSCs) following treatment with PND was analyzed using RNA-seq.

## Results

### RNA sequencing data analysis

**Fig. 1:**
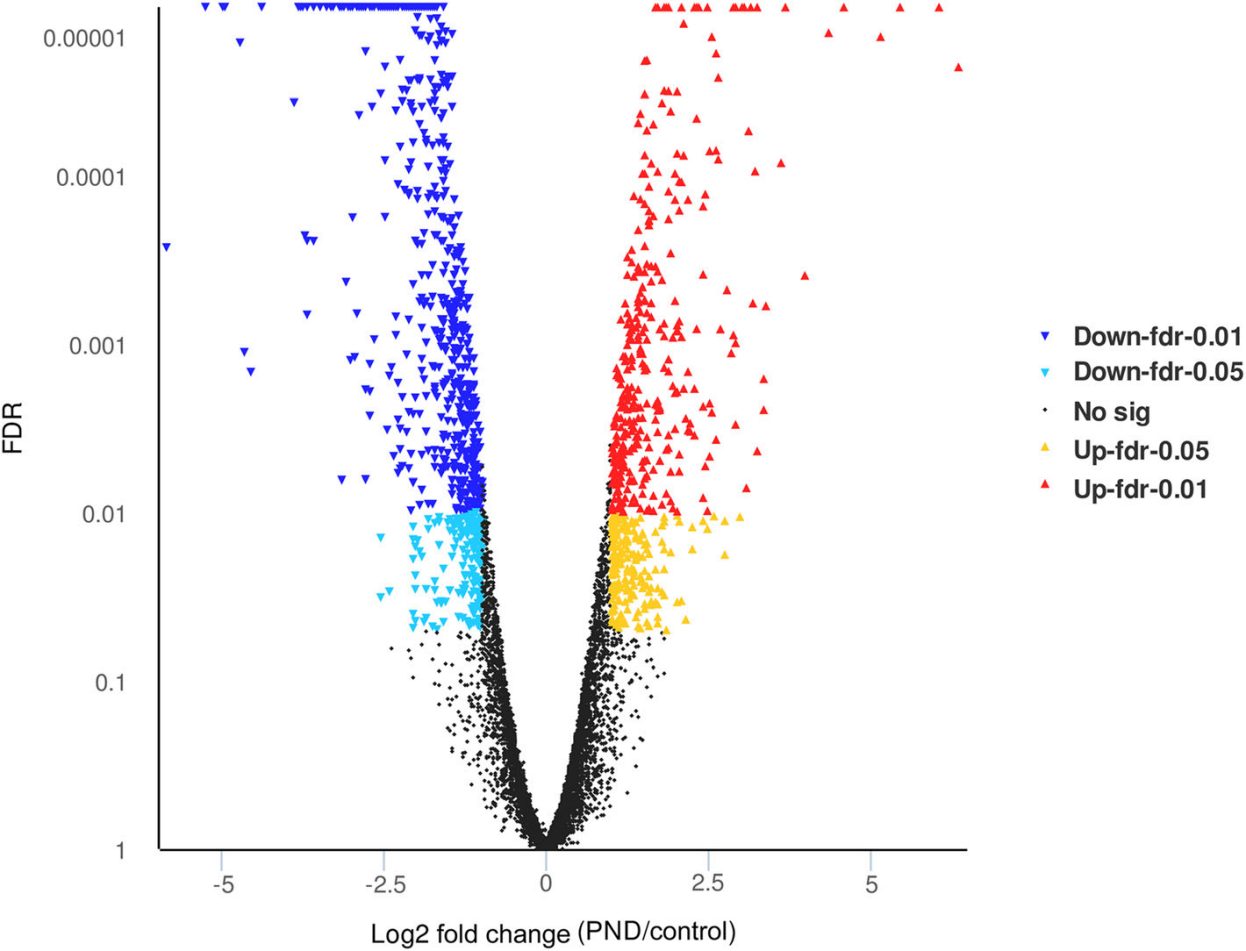
Volcano plot for the differentially expressed genes (DEGs) between control and pyronaridine (PND)-treated groups. The X-axis shows the differences in gene expression (FDR: adjusted *p-value*), while the Y-axis indicates expression changes (log2 fold change) of the genes in different groups. Splashes were for different genes. Black dots, red and yellow dots, and blue and dark blue dots represent genes with no significant discrepancy, significantly upregulated genes, and significantly downregulated genes, respectively

Global gene expression of PND-treated *E. granulosus* PSCs was analyzed using an Illumina platform. The obtained sequences were aligned against *E. granulosus* genome sequences. A total of 60.3 and 58.2 million clean reads were obtained from control and PND groups, respectively (Table 1). The clean reads were mapped to the *E. granulosus* genome scaffold (https://www.ncbi.nlm.nih.gov/genome/?term=Echinococcus+granulosus) reported by Zheng et al. [14]. As shown in a volcano plot (Fig. 1), a total of 1,321 genes were significantly differentially expressed in the PND-treated group compared to the control group, including 541 upregulated and 780 downregulated *E. granulosus* genes.

**Table 1:** Summary of read mapping results of the sequences generated from *E. granulosus* PSCs with or without PND treatment

Sample	Raw reads	Clean reads	Total mapped
Control-1	55,872,838	54,440,078	42,085,096 (77.31 %)
Control-2	58,644,988	57,316,610	46,091,104 (80.41 %)
Control-3	70,831,560	69,270,984	51,122,402 (73.80 %)
PND-1	57,226,520	55,803,992	38,687,749 (72.07 %)
PND-2	61,273,444	59,792,756	36,998,770 (65.32 %)
PND-3	60,595,380	58,885,554	33,587,673 (60.43 %)

### Gene ontology (GO) classification

There were 47 GO terms significantly enriched for differentially expressed genes (DEGs) in PND-treated groups, including 19 biological process terms, 17 cellular component terms, and 11 molecular function terms (Table 2). The most enriched GO terms were associated with cell part, voltage-gated calcium channel complex, binding, voltage-gated calcium channel activity and response to stress.

**Table 2:** GO enrichment analysis of the DEGs of *E. granulosus*

Category^a^	GO term ID	GO term description	*p* value	DEG involved
Cellular component	GO:0044464	Cell part	2.25 × 10^− 7^	320
	GO:0044424	Intracellular part	2.95 × 10^− 6^	272
	GO:0005891	Voltage-gated calcium channel complex	2.53 × 10^− 5^	7
	GO:0034704	Calcium channel complex	6.26 × 10^− 5^	7
	GO:0043229	Intracellular organelle	0.000177	148
	GO:0043226	Organelle	0.000224	148
	GO:0032991	Macromolecular complex	0.000849	176
	GO:0016021	Integral component of membrane	0.000866	135
	GO:0031224	Intrinsic component of membrane	0.000876	135
	GO:0044425	Membrane part	0.0013	157
Molecular function	GO:0005488	Binding	8.54 × 10^− 6^	427
	GO:0005245	Voltage-gated calcium channel activity	2.53 × 10^− 5^	7
	GO:0097159	Organic cyclic compound binding	0.000336	286
	GO:1,901,363	Heterocyclic compound binding	0.000336	286
	GO:0004930	G-protein coupled receptor activity	0.000512	19
	GO:0003676	Nucleic acid binding	0.000647	169
	GO:0043565	Sequence-specific DNA binding	0.000827	36
	GO:0043167	Ion binding	0.000974	278
	GO:0043169	Cation binding	0.00201	158
	GO:0004872	Receptor activity	0.00227	35
Biological process	GO:0006950	Response to stress	9.19 × 10^− 6^	39
	GO:0050896	Response to stimulus	2.64 × 10^− 5^	40
	GO:0006355	Regulation of transcription, DNA-templated	0.000521	85
	GO:0034765	Regulation of ion transmembrane transport	0.00192	9
	GO:0034762	Regulation of transmembrane transport	0.00192	9
	GO:0051049	Regulation of transport	0.00335	10
	GO:0034728	Nucleosome organization	0.00396	11
	GO:0006334	Nucleosome assembly	0.00396	11
	GO:0065004	Protein-DNA complex assembly	0.00396	11
	GO:0071824	Protein-DNA complex subunit organization	0.00396	11

^a^Top 10 terms for each category

### Biochemical pathway

**Fig. 2:**
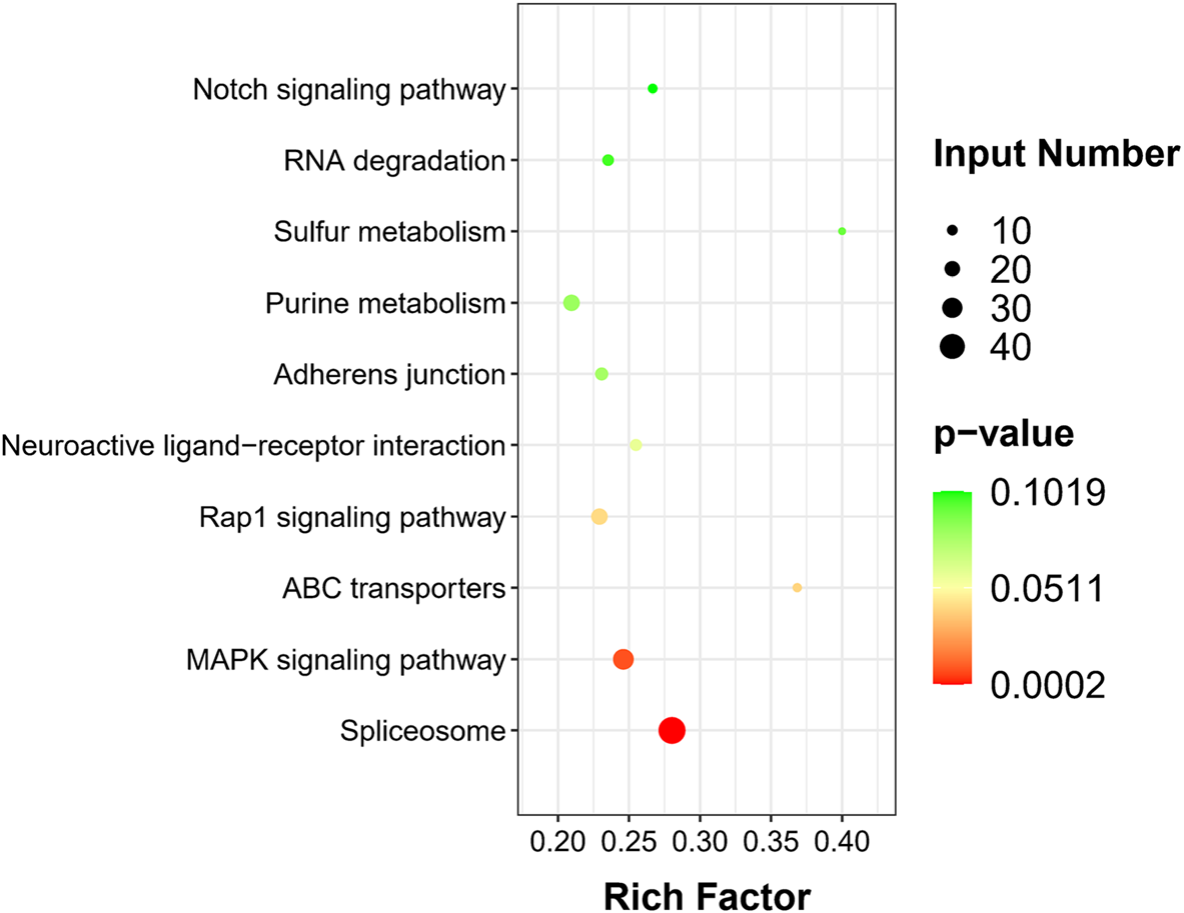
Scatter plot showing the 10 most enriched KEGG pathways of *E. granulosus* induced by PND. The Y-axis label represents the distinct KEGG pathways, and the X-axis label represents the rich factor. The rich factor refers to the ratio of the DEGs annotated in the pathway versus the total number of genes annotated. Dot size is positively correlated to the number of DEGs. The colors of the dots represent the *p* values for the enrichment. Red indicates high enrichment, while green indicates low enrichment

The DEGs were also mapped to five KEGG subsystems, including environmental information processing, genetic information processing, organismal systems, metabolism, and cellular processes. The most significantly enriched 10 pathways are shown in Fig. 2. Spliceosome, mitogen-activated protein kinase (MAPK) signaling pathway and ATP-binding cassette (ABC) transporters were the top three significantly enriched pathways (Figs. S1, S2, S3). PND treatment resulted in significant changes in seven ABC transporter genes (Table 3), and three of them (EGR_00511, EGR_00512 and EGR_01347) are involved in MDR, coding MDR transporters and MDR-associated proteins. In addition, we found that PND treatment induced a significant downregulation in a large number of HSP genes including heat shock proteins (HSPs) and heat shock constitutive cognates (HSCs) (Table 4). Most of the genes are of HSP70 family and involved in the top two enriched pathways. HSP70 is a highly expanded gene family in *Echinococcus* spp. Among the 19 differentially expressed HSP genes, six genes belong to *Echinococcus*-specific expansion of HSP70 (Table 4).

**Table 3:** Differentially expressed ABC transporter genes following the PND treatment

Gene ID	Log2 fold change (PND/control)	ABC transporter subfamily	MDR related
EGR_07315	1.97	ABCA	
EGR_07316	2.07	ABCA	
EGR_07314	2.03	ABCA	
EGR_00512	2.10	ABCB	√
EGR_00511	-2.27	ABCB	√
EGR_01347	1.23	ABCC	√
EGR_02590	1.01	ABCG	

**Table 4:** Differentially expressed HSP genes following the PND treatment

Gene ID	Log2 fold change (PND/control)	NR_Description [*Echinococcus granulosus*]	Spliceosome	MAPK signaling pathway	HSP70 family	*Echinococcus*- specific expansion
EGR_04534	-3.81	Heat shock cognate protein	✓	✓	✓	✓
EGR_11004	-3.29	Heat shock cognate protein	✓	✓	✓	
EGR_06252	-3.77	Heat shock cognate protein	✓	✓	✓	
EGR_08691	-4.96	Heat shock protein 70	✓	✓	✓	
EGR_10493	-3.06	Heat shock cognate protein	✓	✓	✓	✓
EGR_09649	-4.73	Heat shock cognate protein	✓	✓	✓	✓
EGR_10437	-2.8	Heat shock cognate protein	✓	✓	✓	
EGR_09650	-3.88	Heat shock cognate protein	✓	✓	✓	
EGR_10562	2.45	Heat shock protein			✓	
EGR_11188	-5.86	Heat shock protein 70	✓	✓	✓	✓
EGR_03078	1.75	Small heat shock protein p36				
EGR_04903	-1.47	Heat shock cognate protein	✓	✓	✓	
EGR_03136	-1.45	Heat shock protein				
EGR_09244	-2.31	Heat shock cognate protein	✓	✓	✓	✓
EGR_00589	-2.71	Heat shock protein beta-11				
EGR_07753	-2.1	Heat shock cognate protein	✓	✓	✓	
EGR_05222	1.93	Heat shock protein 70	✓	✓	✓	✓
EGR_07332	-1.33	Heat shock cognate protein	✓	✓	✓	
EGR_09751	-1.03	Heat shock 10 kda protein 1				

### Validation of key DEGs by qRT-PCR

**Fig. 3:**
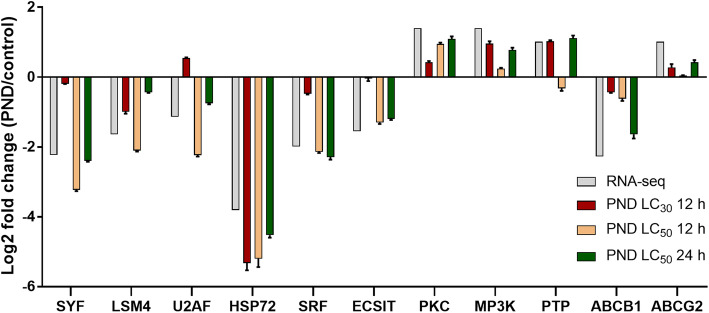
Verification of the RNA-seq data by qRT-PCR. Bars represent the mean fold changes in the expression of eleven genes in *E. granulosus* PSCs treated with PND at the concentrations of LC_30_ (30.6 µM) or LC_50_ (49.0 µM) for 12 or 24 h compared with non-treated PSCs. PSCs used for RNA-seq were treated with PND at the concentration of LC_50_ (49.0 µM) for 24 h

To validate the results of transcriptome sequencing, quantitative PCR (qRT-PCR) was used for confirmation of eleven representative genes selected from the top three most enriched KEGG pathways, including HSP72, SRF, ECSIT, PKC, MP3K and PTP from the MAPK signaling pathway, SYF, LSM4 and U2AF from spliceosome, and ABCB1 and ABCG2 from ABC transporters (Fig. 3). The qRT-PCR expression patterns of nine out of eleven DEGs were in agreement with the results of the transcriptome analysis, despite the variation of drug concentration or treatment time.

### The protein levels of ***Eg***MKK1 and ***Eg***MKK2

**Fig. 4:**
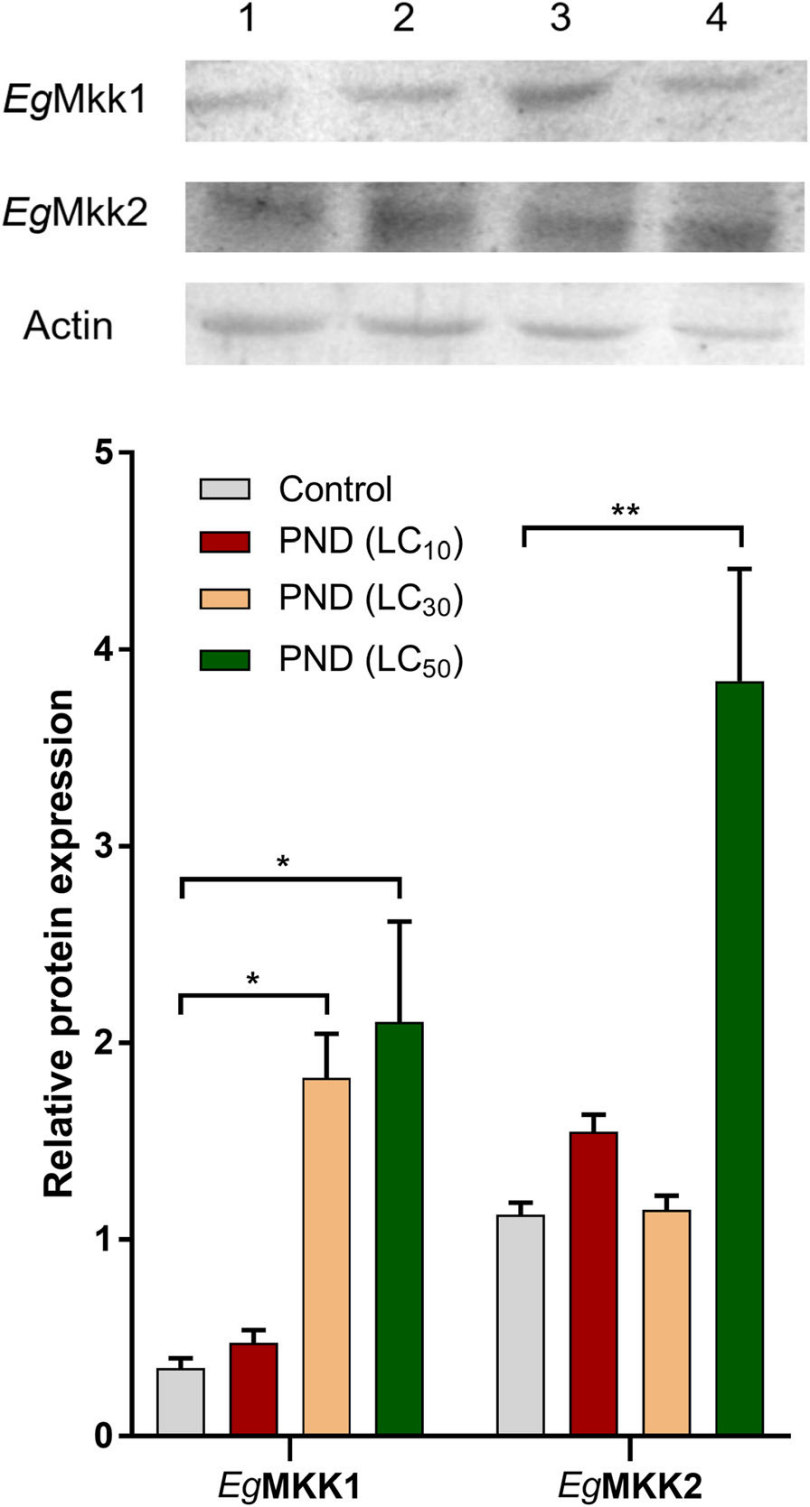
Western blot analysis of the effect of PND on *Eg*MKK1 and *Eg*MKK2. Protein expression levels of *Eg*MKK1 and *Eg*MKK2 in PSCs without (control, lane 1) or with the treatment of PND at the concentrations of LC_10_ (9.9 µM, lane 2), LC_30_ (30.6 µM, lane 3) and LC_50_ (49.0 µM, lane 4, identical treatment to that of RNA-seq samples) for 24 h. *p < 0.05, **p < 0.01, compared to control

To further evaluate the changes of key members of MAPK cascades, we determined the protein levels of *Eg*MKK1 (MKK3/6-like) and *Eg*MKK2 (MEK1/2-like) by Western blotting. As shown in Fig. 4, generally, PND treatment upregulated the protein levels of both *Eg*MKK1 and *Eg*MKK2 in a dose-dependent pattern. For *Eg*MKK1, a significant elevation was observed in PSCs following the treatment of PND at the concentrations of LC_30_ and LC_50_ compared with the control group (*p* < 0.05), while in the case of *Eg*MKK2, it was the treatment of LC_50_ (*p* < 0.01).

## Discussion

CE is a neglected disease that has remained “unattractive” to pharmaceutical companies, considering that, in the last few decades, no alternative drugs have been approved for treating it, although some efforts have been made [15–17]. In our previous study, PND, an approved antimalarial drug, was repurposed as an anti-CE candidate. PND killed 100 % of the cysts in a mouse infection model by intraperitoneal injection at 57 mg/kg/day for three days. When administered orally with a regimen of 57 mg/kg/day × 30 days, it produced 90.7 % cyst mortality, showing that PND is much more effective than albendazole (22.2 % cyst mortality at 50 mg/kg/day), the only anti-CE drug recommended by WHO [8]. However, the anti-parasitic mechanism of PND remains unclear. In this study, RNA-seq technology was used to explore the genes affected by PND on *E. granulosus*.

Using a suitable low dose, our study showed that PND treatment induced changes in the expression of a large number of genes, including 541 *E. granulosus* genes upregulated and 780 downregulated, which demonstrates that PND effectively targets *E. granulosus* PSCs. GO enrichment and KEGG analyses revealed that the significantly PND-altered biological processes and pathways were associated with a wide range of cellular components, biological processes, and metabolic pathways, including cellular structures and signaling pathways.

The MAPK cascade is an evolutionarily conserved signal transduction pathway that transmits and converts many extracellular signals by three consecutive phosphorylation events. MAPK pathways are implicated in several cellular processes, including proliferation, differentiation, apoptosis, inflammation, and stress response [18–20]. According to KEGG enrichment analysis, the MAPK pathway comes in second in the top-changed pathways affected by PND. In the last decade, a few components of the MAPK pathway have been identified in *E. granulosus*, including *Eg*p38, *Eg*ERK, *Eg*MKK1 and *Eg*MKK2 [21–23]. Meanwhile, some MAPK inhibitors (e.g., sorafenib, U0126, SB202190) were found to effectively kill *E. granulosus in vitro* and/or *in vivo* [21, 24, 25], proving that the key kinases could be used as potential targets for anti-CE drug development. Following the exposure of PND, a direct effect on the gene levels of the key nodes of the MAPK pathway was not observed. We speculate that, rather than specifically targeting one key node, PND likely had a general impact on the whole pathway, which was demonstrated by the significantly elevated protein levels of *Eg*MKK1 and *Eg*MKK2.

ABC transporters are transmembrane proteins that actively mediate the translocation of a wide variety of molecules across the cell membrane, including drugs. A subset of ABC transporters is closely linked to MDR, e.g. the best-characterized multidrug transporter Pgp (ABCB1/MDR1). ABC multidrug transporters have been implicated in drug resistance in several parasites [26, 27]. In the genome of *E. granulosus*, 22 putative ABC transporters were identified and could be classified into six subfamilies [28]. In this study, PND treatment induced significant changes in seven ABC transporter genes (Table 3), and three of them (EGR_00511, EGR_00512 and EGR_01347) are involved in MDR, coding MDR transporters and MDR-associated proteins. Usually, anti-parasitic drug treatment would result in increased/over expression of ABC transporters, especially MDR transporters, to remove or exclude xenotoxins from cells to guard the normal cellular physiology [29–31]. While in this study, after the PND treatment, an ABCB gene (EGR_00511) was significantly downregulated (validated by qRT-PCR, Fig. 3). This indicates that, besides the strong protoscolecidal ability, PND could also negatively regulate the expression of *E. granulosus* MDR transporter to favor its retention in PSC tissues as an add-on effect. It inspires us that future research efforts could be geared towards the combination of MDR modulators and current anthelmintics to enhance drug susceptibility.

In addition, we found that PND downregulated several HSP genes in *E. granulosus* PSC. HSPs are originally identified because of their roles in response to heat shock (or other stressors) and these molecules are also molecular chaperones involved in protein folding and maturation [32]. Some HSPs (such as heat shock cognate proteins, HSCs) are constitutively expressed in cells, and serve vital functions in cell metabolism maintenance. We showed that PND treatment induced a significant downregulation in a large number of HSP genes, indicating a close relationship between the drug and HSPs. The differentially expressed HSP genes (Table 4) included five downregulated and three upregulated heat shock proteins and also eleven downregulated constitutive cognates, indicating that not all the DEGs observed in transcriptome analysis were necessarily induced by stress (e.g. an external stimulus caused by PND drug treatment). In addition, most of the HSP genes are of the HSP70 family. HSP70 is a highly expanded gene family in *E. granulosus* [14, 33], and it has been found that some HSP70s may be non-functional transcribed pseudogenes [34]. Through orthology search, six differentially expressed HSP genes were identified to belong to *Echinococcus*-specific expansion of HSP70 (Table 4). HSPs are implicated in the cause and progression of various diseases, such as infections [35], cancer [36, 37], and neurodegeneration [38, 39]. In parasites, such as *Plasmodium* spp. [40], *Leishmania* spp. [41], and *Trypanosoma* spp. [42], HSPs have been investigated as potential drug targets. Some of the HSP genes have already been identified and studied in *E. granulosus* [43, 44]. The results here reported motivate us to study the relationships of PND and HSPs further.

## Conclusions

In this study, the transcriptome landscape of *E. granulosus* PSCs treated with PND was characterized. This allowed the identification of 1,321 DEGs, some of which were found to exhibit great influence on various life processes of *E. granulosus*, including MAPK pathway, ABC transporters and HSPs. These findings provide valuable genetic data to facilitate future studies toward understanding the anti-parasitic mechanism of PND.

## Methods

### Drug treatment of ***E. granulosus*** PSCs

*E. granulosus sensu stricto* PSCs were aspirated from echinococcal cysts of naturally infected sheep livers collected from a slaughterhouse in Urumqi, China [15]. PSCs were treated with 1 % pepsin in saline at 37 °C for 30 min, with the pH adjusted to 3.0. After three washes with PBS, the PSCs were cultured in RPMI 1640 culture medium (Gibco, cat#31,800,022) containing 10 % fetal bovine serum (Gibco, cat#10,099,141 C) and antibiotics (100 U/mL penicillin and 100 µg/mL streptomycin, Gibco, cat#15140-122) in a CO_2_ (5 %) incubator at 37 °C. PND tetraphosphate was synthesized in-house [8] and dissolved in PBS to prepare a drug solution. Viable PSCs were aliquoted in a 24-well plate with each well-containing 2,100 PSCs. The wells were randomly divided into two groups. For RNA-seq, the PND group was treated with a PND solution at a final concentration of 49.0 µM (LC_50_), and the control group received an equal volume of PBS. Each group included three biological replicates. After incubation for 24 h, the treated and control PSCs were washed with PBS and frozen in liquid nitrogen quickly, then stored at -80 °C. For qRT-PCR and western blot analyses, the PSCs were treated with PND at the final drug concentrations of 49.0 µM (LC_50_), 30.6 µM (LC_30_) or 9.9 µM (LC_10_) for 12 or 24 h.

### RNA extraction and cDNA library construction

Total RNA (5 µg) extracted from each sample using TRIzol® Reagent (Invitrogen, cat#15,596,026) at 4 °C was used for RNA-seq analysis. Then, RNA quality was further assessed by the Agilent 2100 Bioanalyser (Agilent Technologies, Santa Clara, CA, USA) and quantified using the NanoDrop spectrophotometer (ND-2000, NanoDrop Technologies). Adapter-modified fragments were selected using gel purification and PCR amplified to create the final cDNA library prepared following the TruSeq™ RNA sample preparation Kit from Illumina HiSeq 4000 (Illumina, San Diego, CA, USA). The Illumina HiSeqxten sequenced the paired-end RNA-seq sequencing library (2 × 150 bp read length, San Diego, CA, USA).

### RNA-seq bioinformatics analysis

The raw reads were subjected to adapter trimming and low-quality filtering using SeqPrep (https://github.com/jstjohn/SeqPrep) and Sickle (https://github.com/najoshi/sickle) with default parameters. The high-quality clean reads were aligned to the reference genome using the HIASAT (https://ccb.jhu.edu/software/hisat2/index.shtml) software. The DEGs between control and PND-treated PSCs were identified based on fragments per kilobases per million reads (FPKM) using the Transcripts Per Million reads (TPM) method. DESeq2 (http://bioconductor.org/packages/stats/bioc/DESeq2/) was used to identify differential expression analysis. Gene expression with log2 fold change ≥ 1 or ≤ − 1, and differences in expression with an adjusted *p-value* < 0.05 were considered to be significant. In addition, the GO and KEGG databases were explored to identify which DEGs were significantly enriched in GO terms and KEGG pathways. GO functional enrichment was carried out by Goatools (https://github.com/tanghaibao/Goatools) [45].

### qRT-PCR assay

Eleven representative genes (four upregulated genes: PKC, MP3K, PTP and ABCG2; nine downregulated genes: HSP72, ECSIT, SRF, SYF, LSM4, U2AF, and ABCB1) were selected from the top three enriched pathways, and their gene expression levels in the control and PND-treated groups were evaluated. GAPDH was used as an endogenous control. Gene expression was quantified with SYBR Green Master (Takara, cat#RR820A, Dalian, China). The primers are shown in Table S1.

### Western blot analysis

The rabbit anti*-Eg*MKK1/anti*-Eg*MKK2 serums are generous gifts from Dr. Chuanshan Zhang, the First Affiliated Hospital of Xinjiang Medical University. Western blot analyses of *Eg*MKK1 and *Eg*MKK2 were performed as previously described [21]. β-Actin served as a loading control.

## Supplementary Information

**Additional file 1:Figure S1** Spliceosome pathway

**Additional file 2:Figure S2** MAPK signaling pathway

**Additional file 3:Figure S3** ABC transporters

**Additional file 4: Table S1** Primer sequences used for qPCR analysis

**Additional file 5:Table S2** All genes with RPKM values and FDRs

**Additional file 6: Table S3** Enriched GO terms of DEGs

**Additional file 7:Table S4** Enriched KEGG pathways of DEGs

## Data Availability

The RNA-seq data obtained in this study were deposited in the National Center for Biotechnology Information (NCBI) Sequence Read Archive (SRA) database (https://www.ncbi.nlm.nih.gov/sra) under accession number of PRJNA667188.

## Abbreviations

ABC: ATP-binding cassette
CE: Cystic echinococcosis
DEGs: Differentially expressed genes
HSPs: Heat shock proteins
KEGG: Kyoto encyclopedia of genes and genomes
GO: Gene ontology
MAPK: Mitogen-activated protein kinase
MDR: Multi-drug‐resistance
PND: Pyronaridine
PSCs: Protoscoleces
qPCR: Quantitative PCR
RNA-seq: RNA sequencing
