# Supplementary figures and images for: Transcriptome analysis uncovers the key pathways and candidate genes related to the treatment of Echinococcus granulosus protoscoleces with the repurposed drug pyronaridine
Source: BMC Genomics. 2021 Jul 13;22:534. doi: 10.1186/s12864-021-07875-w (PMC8276484; doi:10.1186/s12864-021-07875-w)

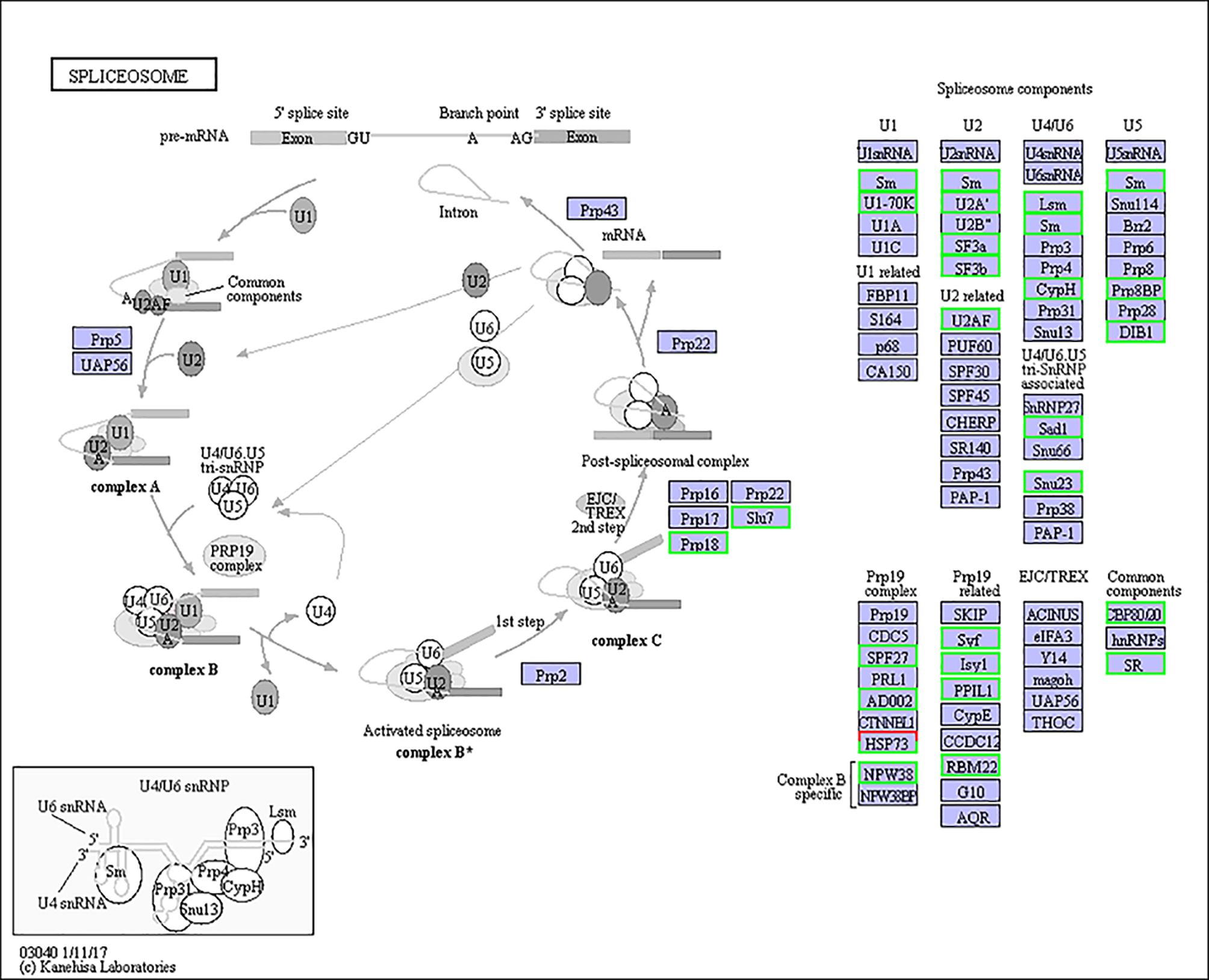

Supplement: Supplementary file 1 — Additional file 1:Figure S1 Spliceosome pathway [file 12864_2021_7875_MOESM1_ESM.png]

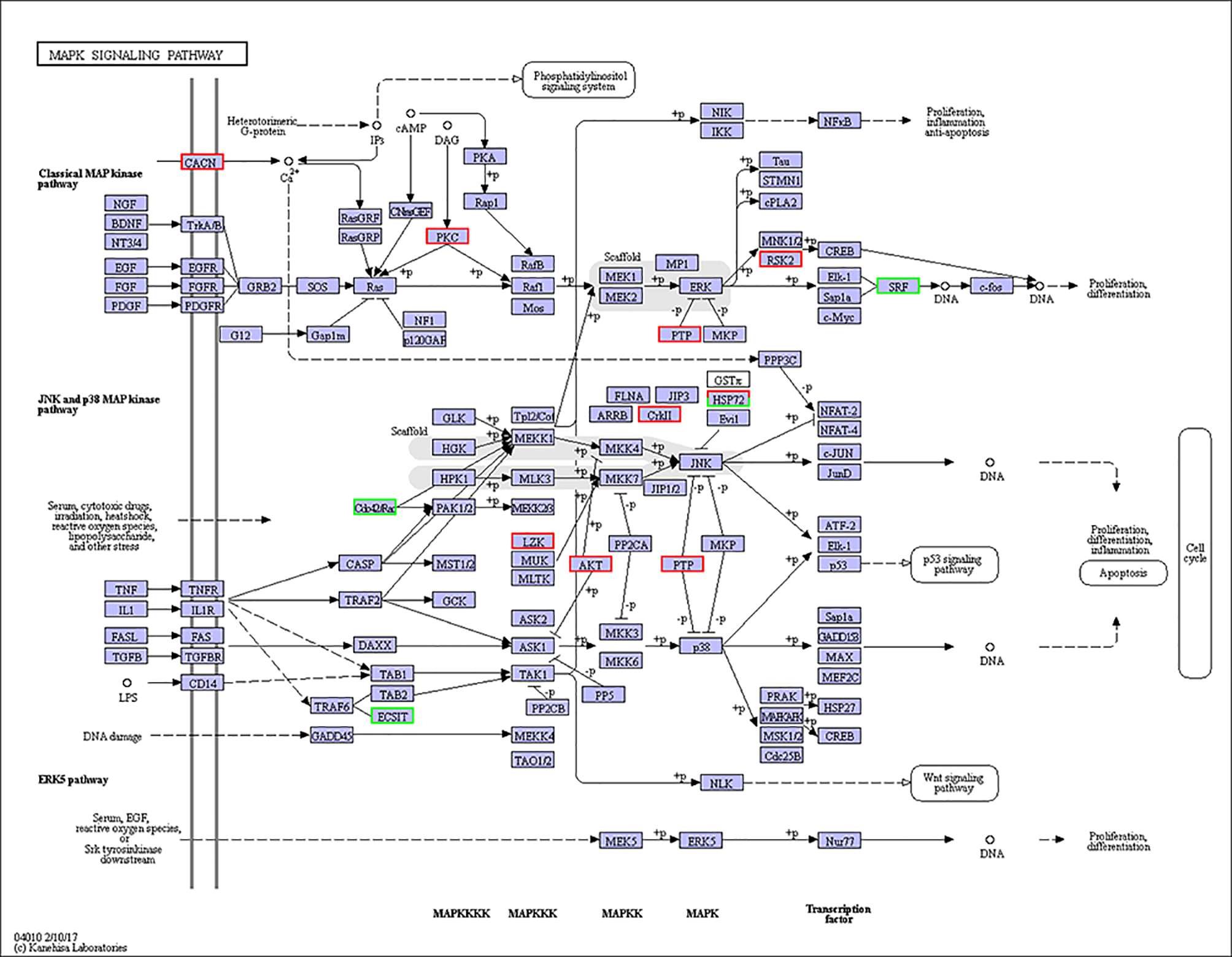

Supplement: Supplementary file 2 — Additional file 2:Figure S2 MAPK signaling pathway [file 12864_2021_7875_MOESM2_ESM.png]

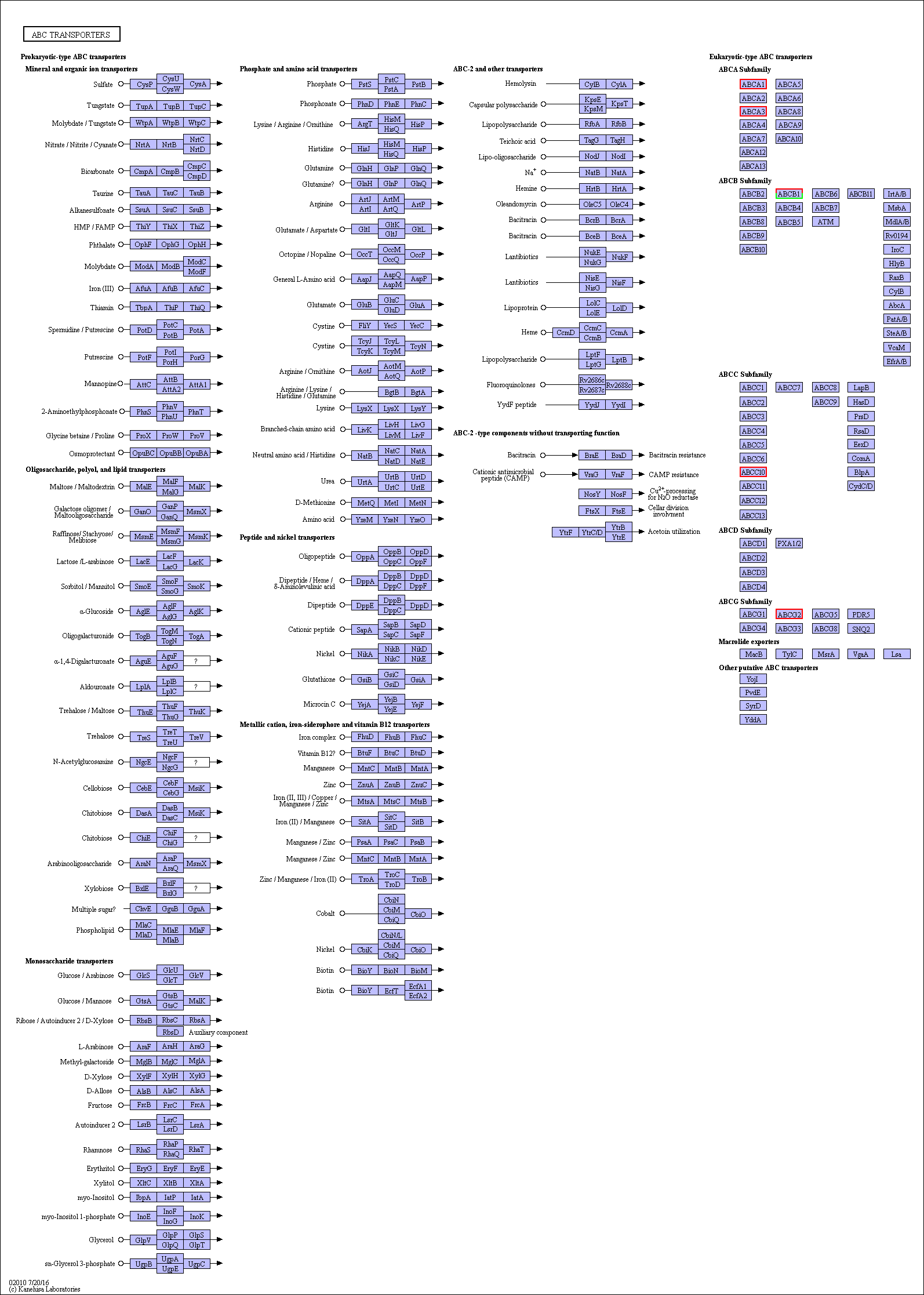

Supplement: Supplementary file 3 — Additional file 3:Figure S3 ABC transporters [file 12864_2021_7875_MOESM3_ESM.png]
