# Supplementary material for: Transcriptome analysis uncovers the key pathways and candidate genes related to the treatment of Echinococcus granulosus protoscoleces with the repurposed drug pyronaridine
Source: BMC Genomics. 2021 Jul 13;22:534. doi: 10.1186/s12864-021-07875-w (PMC8276484; doi:10.1186/s12864-021-07875-w)
Supplement: Supplementary file 4 — Additional file 4: Table S1 Primer sequences used for qPCR analysis [file 12864_2021_7875_MOESM4_ESM.doc]

Table S1 Primer sequences used for qPCR analysis

| Gene | Forward primer | Reverse primer | Gene ID |
| --- | --- | --- | --- |
| RFX | CTTCAAATCGCTGTGCGTCG | GGGTATCCGAATGGTCGTGG | EGR_03082 |
| PKC | TGTTGGCTCTGCAGCAAAAG | ATCAGGTCGCCACCGTTAAT | EGR_03545 |
| MP3K | TGCCTTGGCCCTTTCTGTAG | ACGTCTCTCCTTCTCCGACA | EGR_08017 |
| SRF | GGGGCAAGCAGAAGATTCCT | AAGAAGAAGCACCTCGGCTC | EGR_06766 |
| PTP | CGAGGGTTGTTCTTCCTGCT | CGTTGATCGTGTGCGACATC | EGR_01369 |
| ECSIT | CAATGTCGAATGCGCTGGAG | CTGGGAGAGACACGGTAGGA | EGR_02787 |
| SYF | CCGCGCCAAAAATCTGAACA | TTCTTATGAGAGGCGTCGGC | EGR_07536 |
| LSM4 | CGCGATGGTGACAGGTTTTG | GTACCCTCGACGCTTGCATA | EGR_04393 |
| U2AF | GCTACGCCTTCTGCGAGTAT | TAAGGCCAGGCACCTGAATG | EGR_02775 |
| HSP72 | TGCTCTACCGAAGTCGATGC | ACCGCCTCAGCGCTATTATC | EGR_04534 |
| CTSL | ACGGCTCGAGATGGAAAGTG | TTCTCCGGCACTTTGACGAA | EGR_08123 |
| CALR | AATACCACAGAGTCGGCACG | ATGTATCCACCGGCACAGTC | EGR_08107 |
| ABCA3 | TATGGCCTTTCACGAACCTC | ACATATTGTTCACCGCGACA | EGR_07314 |
| ABCB1 | GTGGTATCGCTGGTGAGGT | TCCAGTTCGCTCAGCCTTAT | EGR_00511 |
| ABCG2 | ATTTTGATGGGAACGCTGAC | TTCTCACTCGTTTCGGCTTT | EGR_02590 |
| GAPDH | AGGTCGGTGTGAACGGATTTG | TGTAGACCATGTAGTTGAGGTCA | NM_001289726.1 |

EGR: *E. granulosus ss*
